# Intact Auditory Cortical Cross-Frequency Coupling in Early and Chronic Schizophrenia

**DOI:** 10.3389/fpsyt.2020.00507

**Published:** 2020-06-04

**Authors:** Nicholas Murphy, Nithya Ramakrishnan, Christopher P. Walker, Nicola R. Polizzotto, Raymond Y. Cho

**Affiliations:** ^1^Psychiatry and Behavioral Sciences, Baylor College of Medicine, Houston, TX, United States; ^2^Research Service Line, Michael E. DeBakey Veterans Affairs Medical Center, Houston, TX, United States; ^3^Department of Psychiatry, University of North Carolina at Chapel Hill, Chapel Hill, NC, United States; ^4^Psychiatry and Behavioral Sciences, University of Texas Health Science Center at Houston, Houston, TX, United States; ^5^Menninger Clinic, Houston, TX, United States

**Keywords:** schziophrenia, ASSR = auditory steady-state response, phase amplitude coupling, biomarker, Cross Frequency Coupling (CFC), magnetoencephalagraphy (MEG)

## Abstract

**Background:**

Previous work has identified a hierarchical organization of neural oscillations that supports performance of complex cognitive and perceptual tasks, and can be indexed with phase-amplitude coupling (PAC) between low- and high-frequency oscillations. Our aim was to employ enhanced source localization afforded by magnetoencephalography (MEG) to expand on earlier reports of intact auditory cortical PAC in schizophrenia and to investigate how PAC may evolve over the early and chronic phases of the illness.

**Methods:**

Individuals with early schizophrenia (n=12) (≤5 years of illness duration), chronic schizophrenia (n=16) (>5 years of illness duration) and healthy comparators (n = 17) performed the auditory steady state response (ASSR) to 40, 30, and 20 Hz stimuli during MEG recordings. We estimated amplitude and PAC on the MEG ASSR source localized to the auditory cortices.

**Results:**

Gamma amplitude during 40-Hz ASSR exhibited a significant group by hemisphere interaction, with both patient groups showing reduced right hemisphere amplitude and no overall lateralization in contrast to the right hemisphere lateralization demonstrated in controls. We found significant PAC in the right auditory cortex during the 40-Hz entrainment condition relative to baseline, however, PAC did not differ significantly between groups.

**Conclusions:**

In the current study, we demonstrated an apparent sparing of ASSR-related PAC across phases of the illness, in contrast with impaired cortical gamma oscillation amplitudes. The distinction between our PAC and evoked ASSR findings supports the notion of separate but interacting circuits for the generation and maintenance of sensory gamma oscillations. The apparent sparing of PAC in both early and chronic schizophrenia patients could imply that the neuropathology of schizophrenia differentially affects these mechanisms across different stages of the disease. Future studies should investigate the distinction between PAC during passive tasks and more cognitively demanding task such as working memory so that we can begin to understand the influence of schizophrenia neuropathology on the larger framework for modulating neurocomputational capacity.

## Introduction

The neurophysiological basis of sensory and cognitive impairments in schizophrenia has been intensely investigated in order to aid biomarker development for early illness detection and inform the development of novel treatment approaches. Cortical oscillations are widely reported to be disturbed in the illness, and lend insight regarding the integrity of neural network activity related to perception and cognition (1). Core cognitive disturbances, such as impaired cognitive control, are associated with gamma (30–50 Hz) oscillatory impairments, and reflect the disrupted functioning of pyramidal cells and parvalbumin (PV) interneurons in schizophrenia (2–7). Electrophysiological measures such as electroencephalography (EEG) and magnetoencephalography (MEG) can index the entrainment of neural circuits to externally driven stimulation, eliciting a steady-state response that resonates at the stimulating frequency and is suggested to reflect the synchronization of endogenous oscillations (8). On the grounds of well-established auditory perceptual (9) and cortical (10) disturbances in schizophrenia, the auditory steady state response (ASSR) paradigm has emerged as a reliable tool for assessing the physiological (11–14) and pharmacological elements of gamma oscillatory disturbance in the illness (11, 15).

As our understanding of gamma dysfunctions has grown, there has been a push to investigate more detailed mechanics that describe how gamma oscillations act as part of a complex system. A crucial aspect of cortical oscillations that has received growing attention in schizophrenia research is phase-amplitude coupling (PAC). PAC is a form of crosstalk between different frequency oscillations, with the high-frequency amplitude being modulated by the phase of a lower frequency oscillation. In the human auditory cortex, PAC in response to passive and active auditory perception is primarily observed between theta (4–7 Hz) phase and gamma (30–50 Hz) amplitude (16–22). The physiological purpose of PAC is broadly thought to be enhanced neurocomputational capacity within and between networks in a metabolically efficient manner (23, 24), where coupling is regulated dynamically and at multiple spatial and temporal scales (25, 26).

Interestingly, in contrast with findings of decreased gamma band ASSR in schizophrenia (11), PAC during this paradigm appears to show no appreciable impairments in patients suggesting that there may be relative preservation of the physiologic mechanisms supporting PAC (17, 27). Kirihara and colleagues (27) investigated theta-gamma PAC during the 40-Hz ASSR in chronic schizophrenia patients and reported that despite reduced gamma phase coherence and increased theta amplitude in the patient group, there were no group differences in PAC strength. The lack of a discernable difference in theta-gamma PAC was recently corroborated by Hirano and colleagues (17) who reported no group differences but found strong left lateralized coupling in controls, but no lateralization was found in schizophrenia patients.

Given the importance of understanding the impairments and, indeed, the relative preservation of mechanisms supporting coordinated neural circuit activation in the illness, the current study further investigates this question and addresses a number of methodologic limitations of prior studies. The study by Kirihara and colleagues (27) limited their investigation to 40 Hz PAC, and so lacked any inference about frequency specificity in their findings. Our study addresses this limitation through examination of multiple stimulation frequencies (20, 30 and 40 Hz). In human adult subjects, ASSR evoked power at 40 Hz is maximal compared to 20 and 30 Hz (28, 29). In schizophrenia patients, while there is a deficit in 40 Hz ASSRs, 30 and 20 Hz ASSRs are mostly unaffected (30–32) with some exceptions (14, 33). By examining 20 and 30 Hz conditions, we are able to determine the frequency specificity of PAC in MEG-ASSR source estimates. Hirano and colleagues (17) examined only the baseline pre-ASSR stimulation period to prevent confounding PAC changes due to changes in power spectra caused by evoked activity (34), thus making any direct inferences regarding the active cortical entrainment period implausible. For this reason, we restricted our PAC analysis to the steady state portion of the trials, employed surrogate data and comparisons with the baseline period, as well as combined this approach with non-parametric statistics (35). To better evaluate source estimates of impairments in ASSR PAC, we additionally employed MEG which has a superior ability over EEG to separate cortical sources due to reduced spatial smearing associated with the measurement of magnetic rather than electrical fields (36). Compared to EEG, which is more sensitive to radial sources, MEG, which is more sensitive to tangential sources, is favorable for localizing the auditory cortex (37–39). Almost no studies to date have explored the changes in ASSR induced PAC over the illness course in schizophrenia patients. The lack of specificity of ASSR deficits to illness stage contrasts with the significant differences in neural signatures between early and chronic schizophrenia patients (40). Early-stage schizophrenia can be characterized by acute effects of NMDA-R hypofunctioning (40, 41), not evidenced in chronic schizophrenia patients, by means of a gradual shift in excitation/inhibition balance that implicates increased glutamatergic neurotransmission in early-onset patients (41, 42). Overall, increased glutamatergic metabolites in early schizophrenia (42) and decreased glutamatergic metabolites in chronic schizophrenia have been found (43). Thus far, most studies for gamma deficits in schizophrenia, by means of 40-Hz ASSR have focused on chronic schizophrenia, with very few examining the pattern of deficits in early-onset, first-episode patients or at-risk populations (8), and even fewer assessing PAC changes. This study aims to map the trajectory of PAC changes over the different illness phases by investigating both early and chronic phase schizophrenia patients.

## Methods

### Participants

We recruited N = 17 healthy controls (HC) (mean, 28.87 ± 5.98 years), N = 12 patients with early stage schizophrenia (EA) (mean, 27.5 ± 6.89 years), and N = 16 patients with chronic stage schizophrenia (CR) (mean, 33.63 ± 6.94 years). There are some preliminary findings which suggest that dysfunctions in neural oscillations and synchronization are present around the onset of schizophrenia but that prolonged medication usage might have varying effects (29). To address this question, we divided patients into early and chronic disease stages based on illness duration which have been proposed to more accurately capture the split between the critical but varied early stages of the disease and the point at which most patients have begun more steady treatment (early phase defined by ≤5 years of illness duration; and chronic, > 5 years) (44, 45). This study was carried out in accordance with the recommendations and approvals of the University of Texas Health Science Center at Houston Institutional Review Board. Written informed consent was given by all participants in accordance with the Declaration of Helsinki. Diagnoses were confirmed by Structured Clinical Interview for DSM-V Disorders. Further cognitive and clinical characterization included assessments of psychopathology using the Scale for the Assessment of Positive Symptoms (SAPS), the Scale for the Assessment of Negative Symptoms (SANS), the seven-point Clinical Global Impression (CGI) Scale, the Global Assessment Functioning (GAF), the Brief Assessment of Cognition Scale (BACS), and the Social Functioning Scale (SFS).

### Task

Auditory click trains (1000 ms) were presented binaurally (ER-3A insert earphones, Etymotic Research, IL, USA) as 40 Hz, 30 Hz, or 20 Hz repetitions of 1 ms duration tones (1000 Hz) using E-Prime software (Psychological Software Tools, PA, USA). To ensure attentional engagement, participants were presented the click trains in the context of an Oddball paradigm with Standard (click train consisting of 1000 Hz tones) vs. Oddball (click train consisting of 2000 Hz tones) stimulus trials. We presented N = 100 trials (85 standard, 15 oddball) as 10 blocks of 10 trials/block. Oddball tone (2000 Hz) placement was randomly assigned across the total number of trials and did not follow a trial-by-trial order. At the end of each set of 10 trials participants were asked to indicate the number of oddball trials that were presented using a response box. Oddball trials were not included in the analyses. The click train conditions were presented sequentially (i.e., all 40 Hz, then all 30 Hz, then all 20 Hz) and counterbalanced across participants to reduce order effects. To reduce wandering gaze and head motion, we required participants to observe a fixation cross displayed at eye level on a monitor in front of them.

### MEG and MRI Recording

Cortical signals were recorded using a 306-channel Elekta Neuromag TRIUX system, and digitally sampled at a rate of 1000 Hz. Head motion was monitored with continuous head position indicator (cHPI) coils co-registered with digitized fiducial points (nasion, bilateral pre-auricular points) and scalp contour. Eye movement was recorded by placing two bipolar electrode pairs to record vertical and horizontal electrooculogram (EOG). Structural MRI was recorded with a Phillips Ingenia 3T scanner using the following parameters: TR = 1630, TI = 0.8 s, TE = 2.48 ms, 8° flip angle (maximizing gray/white contrast), 256 × 256 × 224 acquisition matrixes, FOV = 205 × 205 mm^2^, 0.8-mm slice thickness, yielding isotropic 0.8-mm^3^ voxels.

### MEG Pre-Processing

Our MATLAB and Python code is available at (https://github.com/NikMNclUth/MEG_CFC). MEG data was processed using the MNE Python toolbox (46, 47). To remove artifacts caused by external interference with the cortical field, we applied temporal signal space separation (TSSS) with a 10-s window (48–50). Bad channels were identified and marked prior to this to improve the accuracy of the estimation. Head motion was corrected by subtracting the information provided by cHPI monitoring. The TSSS corrected data were high pass filtered using a 0.1-Hz Butterworth filter, and then entered into extended-infomax independent components analysis (ICA) to identify and remove components representing EOG and electro-cardiogram (ECG) noise. For each stimulus condition, we created epochs of length −1500 to +1500 ms relative to the stimulus onset, and subtracted the mean of the baseline period from the post-stimulus data. Bad epochs were rejected if peak-to-peak amplitude was beyond reasonable limits for that channel type (>= 100e^−12^ fT for magnetometers and 4000e^−13^ fT/cm for gradiometers). A final manual inspection was then performed to review remaining bad epochs.

### Source Localization

**Figure 1:**
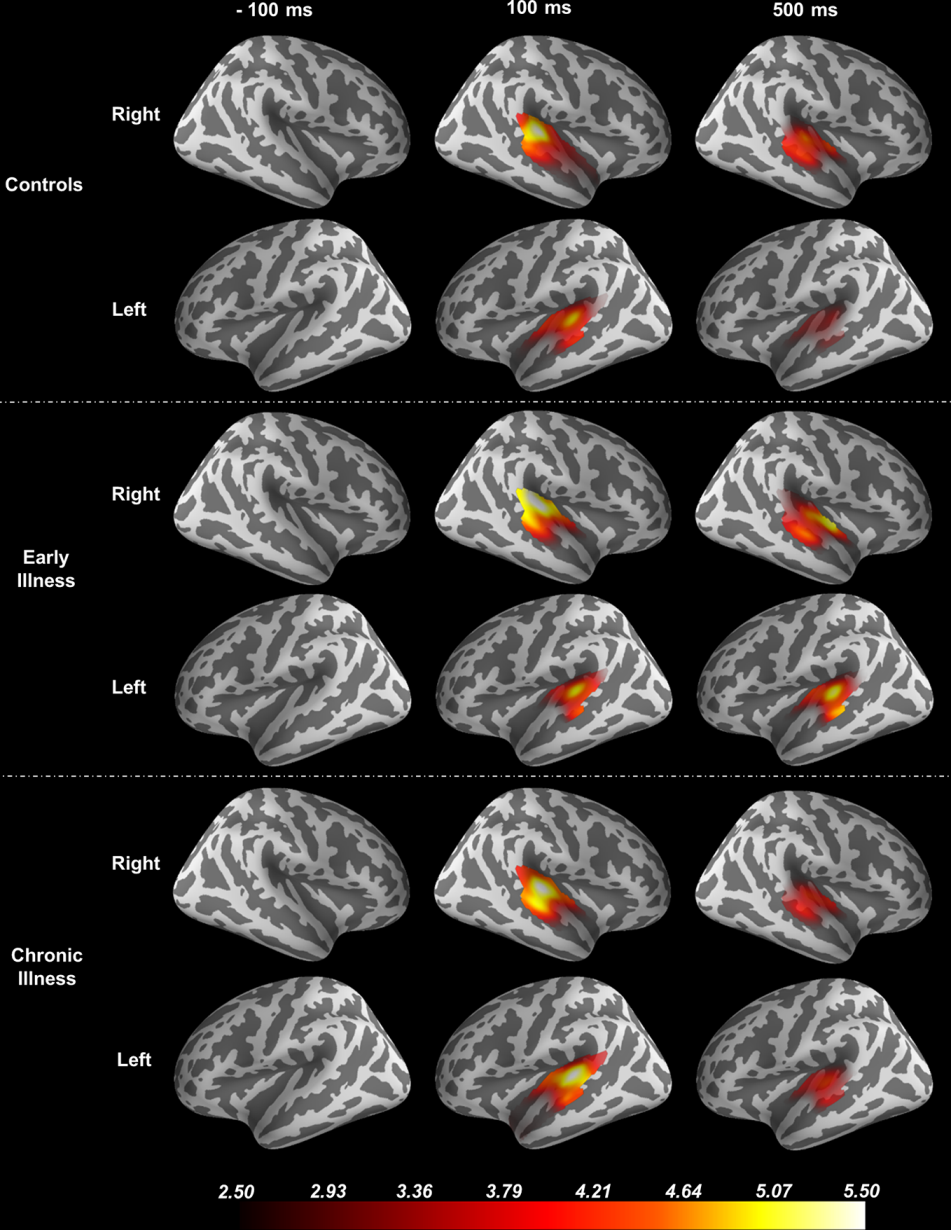
Source Localization of the 40-Hz ASSR evoked data using dSPM. The source estimates are restricted to the anatomical label of bilateral transverse temporal cortex and superior temporal gyrus and remapped (morphed) onto the FreeSurfer average brain.

Using the FreeSurfer package (51, 52), we processed anatomical MRI images using an automatic procedure to obtain the cortical reconstruction (a high-resolution triangulation of the interface between the white and the gray matter) and the inner skull surface (53). We then co-registered the MEG data with the structural MRI by aligning the high-resolution Freesurfer surface model to the MEG data, guided by manually indicating where the fiducial points, digitized at the MEG acquisition, were located on the head surface data. We calculated the forward model using a single compartment boundary element model (BEM) and used the baseline period of the data (−1500 ms to 0 ms relative to the stimulus) to generate a noise covariance matrix. To generate the inverse operator, we defined a surface oriented source space with a depth-weighting coefficient of 0.8. We then estimated source space signals using the dynamic statistical parametric mapping (dSPM) method (54). Regions of interest (ROI) were determined based on the Desikan-Killiany atlas using Freesurfer labels for the combined region of bilateral transverse temporal cortex and superior temporal gyrus to focus the spatial information on the anatomical locus of the ASSR (55). See **Figure 1**. For group-level analysis, the “fsaverage” brain, a template brain model, provided by FreeSurfer (52) was morphed to each individual subject.

### Time–Frequency Analysis

Oscillatory amplitude and inter-trial phase coherence (ITC) were estimated for the average signal across all sources in the functional label, using Morlet wavelets for the frequency range 1 to 60 Hz in a window of −1.5 to 1.5 s. The Morlet wavelets used are described in **Equations 1A, B** where i is the imaginary operator, f is the frequency in Hz, t is the time in seconds, σ is the width of the gaussian, and n is the number of cycles. For wavelet estimation we used n = 10 cycles at all values of f to prioritize frequency resolution. Amplitude estimates were baseline corrected by subtracting the average amplitude from the window of −1300 to −500 ms.

Equation 1A $$W=\;e^{2i\pi ft}e^{\frac{-t^{2}}{2\sigma^{2}}}$$

Equation 1B $$\sigma =\;\frac{n}{2\pi f}$$

**Equation 1. (A)** Time–frequency amplitude estimates for linearly spaced frequencies between 1 and 60 Hz were generated by convolving the evoked time series data with Morlet wavelets. Here *i* is the imaginary operator, *f* represents the frequency of interest, *t* is the time in seconds, *n* is the number of cycles, and σ represents the width of the gaussian. **(B)** For a given wavelet the width of the gaussian used in **(A)** is equal to the number of cycles over the product of 2π*f*.

### Phase-Amplitude Coupling

To estimate PAC we used the method described in Canolty et al. (56). In this approach the amplitude signal is multiplied by the exponent of the phase signal to create a complex composite signal. The mean vector length (MVL) is then derived from the modulus of the mean across trials (see **Equation 2**), where n is the total number of time points, *t* is the Nth time point, *i* represents the imaginary operator, and *Ф* is the phase angle in radians.

Equation 2 $$\mathrm{PAC}=\;\left|n-1\sum_{t=1}^{n}a_{t}e^{i\phi t}\right|$$

**Equation 2**. Phase amplitude coupling is estimated by measuring the mean vector over across the trial dimension at each sample in the time window and then averaging across the time dimension.

Coupling was estimated using Morlet wavelet transforms of the source time series for high frequencies (amplitude; fA) 13 to 60 Hz, and low frequencies (phase; fP) 4 to 12 Hz, using a width of 10 cycles and 1-Hz intervals between frequencies. In addressing frequency-time resolution tradeoffs due to the uncertainty principle, the common methods of devising the bandwidth for high-frequency vs. low-frequency filters (35, 56) suffer from an unequal trade-off between temporal and spectral resolution across bands (34, 57). The use of wavelets in place of conventional filtering offers greater invariance to signal length (58, 59) and addresses the problem of an unequal trade-off between resolutions by increasing the bandwidth of the filter as the peak frequency of the impulse response function increases (57). Coupling was estimated using the steady state portion of the trials (200 ms to 1000 ms) and separately for the baseline portion of the trials (−1300 to −500 ms). To reduce the bias produced by large trial-wise variations in amplitude, the fA signals were normalized using a min-max transform using the values between −1300 and +1300 ms, and the estimated coupling values were transformed to z-scores using the PAC values derived from a distribution of 250 surrogates. We generated surrogate time series by randomly permuting segments of the original signal and then performing the wavelet transform to measure the phase values (60).

For each stimulus condition, and hemisphere, significant instances of coupling were identified by performing a cluster-based permutation t-test (N = 5000 permutations) between the ASSR period and the baseline period using data concatenated across all three groups. The permutation test used routines from the Mass Univariate toolbox (61), employing a one-tailed test (ASSR > Baseline), family-wise error rate of 1%, and a cluster inclusion p-value threshold of 0.01. A cluster was included in the final analysis if its corrected p-value was less than 0.05. To gauge the extent to which the presence of non-sinusoidal oscillations might have biased coupling estimation we calculated the ratio of the time taken for an oscillatory peak to be reached from a trough relative to the time it took to reach a trough from a peak in the fP signals, termed the rise-decay ratio (35). We compared this between stimulation and baseline periods of coupling using a t-test Bonferroni corrected for multiple comparisons.

### Statistical Analysis

Continuous demographic and clinical data were compared using univariate analysis of covariance (ANCOVA), with Bonferroni corrected *post hoc* tests, and controlling for age. Since the lowest group count for gender was less than five, differences in gender distribution were tested using Fisher’s exact test. Amplitude and phase-locking data were analyzed separately for each condition using a mixed design ANCOVA (between-group factors of group, within-group factor of hemisphere) controlling for participant age. We used age as a covariate to control for developmental effects on ASSR gamma synchronization (62), and differences in symptomology based on the age of onset (63–65). The distribution of genders by group is described in the results section. We did not covary for gender due to the uneven distribution and analytical power limitations.

We compared significant PAC clusters between groups using an aligned rank transform Multivariate ANCOVA (66) with a between subjects factor of group and age as a covariate. We used Spearman’s rank correlation coefficient (Spearman’s Rho) to measure any remaining influence of signal amplitude (for fA and fP signals) on PAC strength. Significance across all tests was defined as p <= 0.05.

## Results

### Demographic and Clinical Information

Demographic and clinical information are summarized in **Table 1**. Groups were not matched on age (F (2, 41) = 3.48, p = 0.04, η^2^ = 0.15); the *post hoc* follow-up to our ANOVA revealed that CR were typically older than EA (mean difference = 6.13 years, p = 0.05) but not HC, and that HC and EA were not different. This particular pattern is to be expected based on the illness duration associated with respective staging (early, younger vs. chronic, older). Gender was not equally distributed between groups (HC: 9 males, 8 females; EA: 12 males, 0 females; CR: 13 males, 3 females; Fisher’s exact test *p* = 0.012). In our EA group, all 12 patients reported currently taking antipsychotic medication (1 typical, 11 atypical), and 4 patients reported concurrently taking additional medications (3 on antidepressants, 1 on benzodiazepine). In the CR group, 13 patients reported currently taking antipsychotics (3 typical, 10 atypical). Six patients within the CR group reported concurrently taking other medications (5 on antidepressants, 1 on both benzodiazepine and antidepressant). Total BACS showed a main effect of group (F(3, 40) = 8.5, p < 0.001, η^2^ = 0.11) with no effect of age. Post hoc follow-up indicated both patient groups (EA and CR) having significantly lower BACS scores relative to HC (EA: p = 0.31; CR: p < 0.001) but were not significantly different from each other. Patient groups did not differ significantly on SANS (F(2, 22) = 0.49, p = 0.61, η^2^ = 0.043), or SAPS (F(2, 18) = 0.69, p = 0.51, η^2^ = 0.07).

**Table 1:** Demographic and clinical information.

Item	Controls (N = 17, 8 Female)	Early Illness (N = 12, 0 Female)	Chronic Illness (N = 16, 3 Female)
Age (years)	28.8 ± 5.9	27.5 ± 6.9	33.6 ± 6.9*
Brief Assessment of Cognition (BACS)	51.2 ± 9.2*	39 ± 13*	30.5 ± 13.8*
Age of Onset (years)	–	24.9 ± 5.8	22.1 ± 6.4
Social Functioning Scale (SFS)	–	19.7 ± 3.8	22.7 ± 4.8
Negative Symptoms Scale (SANS)	–	8.6 ± 3.5	8.5 ± 4.5
Positive Symptoms Scale (SAPS)	–	5.2 ± 2.2	6.6 ± 4.2
Clinical Global Impression 1 (CGI1)	–	2.6 ± 0.6	3.4 ± 0.9
Clinical Global Impression 2 (CGI2)	–	5.9 ± 1.6	6.4 ± 1.5
Global Assessment of Functioning (GAF)	–	53.9 ± 13.6	54.7 ± 18.9
Global Functioning Scale (GFS)	–	5.6 ± 1	5.7 ± 1.4

*Significant at p < 0.05.

### Phase-Amplitude Coupling

Cluster analysis revealed three significant patterns of stimulus-related coupling (ASSR > Baseline) between gamma amplitudes (33 Hz to 49 Hz) and delta (4 Hz, cluster mass = 19.74, p = 0.02), theta (6 Hz, cluster mass = 20.94, p = 0.03) and alpha phase (10 Hz, cluster mass = 43.7, p = 0.005) within the right hemisphere source for the 40-Hz entrainment condition. There were no significant clusters for the 30- or 20-Hz conditions (see **Supplement 1**). Using Pillai’s trace there was no significant effects of group membership on cluster coupling strength (V = 0.152, F(6,78) = 1.07, p = 0.39, η2 = 0.07). As a follow-up, we also investigated coupling properties in the baseline period and the difference between ASSR and baseline coupling strength to determine whether any differences in functional activation in response to entrainment might be present. Pillai’s trace revealed that there was a significant main effect of group on coupling strength (V = 0.36, F(6,78) = 2.85, p = 0.014, η2 = 0.18). Univariate follow-up tests revealed that there was a significant effect of group for the theta-gamma (F (2, 40) = 3.77, p = 0.032, η2 = 0.16) and alpha-gamma (F (2, 40) = 5.32, p = 0.009, η2 = 0.21) coupling driving the multivariate test results. Post hoc pairwise comparisons for the univariate tests demonstrated that for theta-gamma coupling there was a marginal increase in baseline coupling strength in the chronic illness group relative to the control group that did not achieve statistical significance (p = 0.054). In addition alpha-gamma coupling in the baseline period was significantly higher in the early illness group than in healthy controls (p = 0.008).To account for potential inflation of the type one error rate by the use of multiple univariate tests we performed a complimentary discriminant analysis, which revealed two discriminant functions. The first explained 62.4% of the variance (canonical R2 = 0.46), while the second explained 37.6% of the variance (canonical R2 = 0.37). Both functions were able to significantly differentiate the treatment groups (combined functions: Λ = 0.68, χ2 (6) = 15.71, p = 0.015; removal of the first function: Λ = 0.86, χ2 (2) = 6.07, p = 0.048). By correlating the outcomes with the discriminant functions we identified that theta-gamma coupling loaded more strongly onto the first function (r = 0.97), whereas alpha-gamma and delta-gamma coupling were loaded more highly onto the second function (ag: r = 0.862; dg: r = 0.29). The relationship of our functions to the group centroids (see **Supplement 1**) showed that function 1 discriminated the control group from the early and chronic illness groups, while function 2 discriminated the early illness group from the control and chronic illness groups. The pattern of group discrimination for the two functions supports the trend seen in the univariate ANOVA *post hoc* tests. When evaluating the resulting differences in PAC between conditions (ASSR-BL) Pillai’s trace did not demonstrate a significant effect of group membership for change in PAC strength between states (V = 0.08, F (6, 78) = 0.51, p = 0.79, η2 = 0.04).

**Figure 2:**
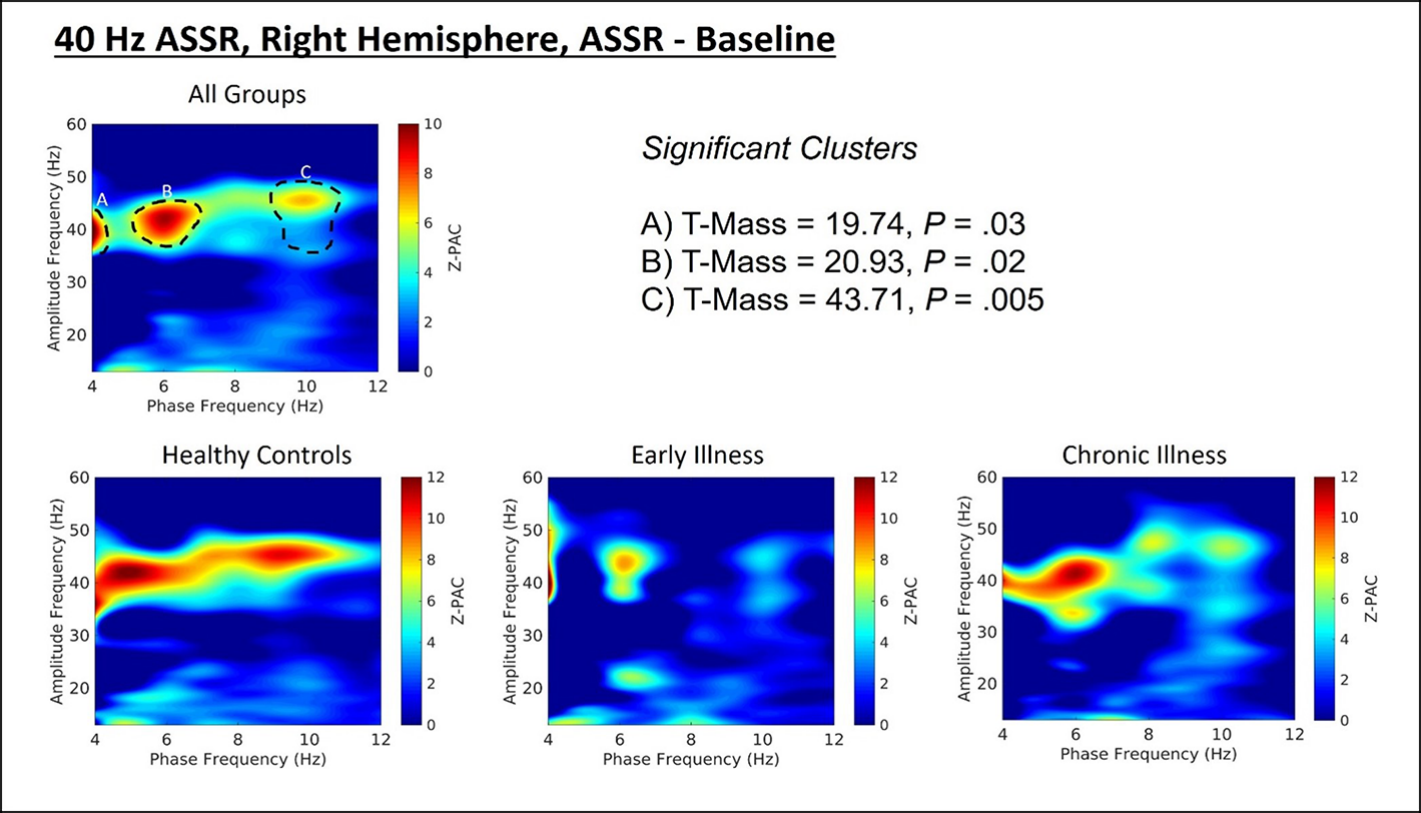
(Top) Right hemisphere Phase-amplitude coupling T-maps demonstrating differences between stimulation and baseline periods of the trial, with subjects pooled across groups. (Bottom) Right hemisphere PAC compared between groups.

**Figure 3:**
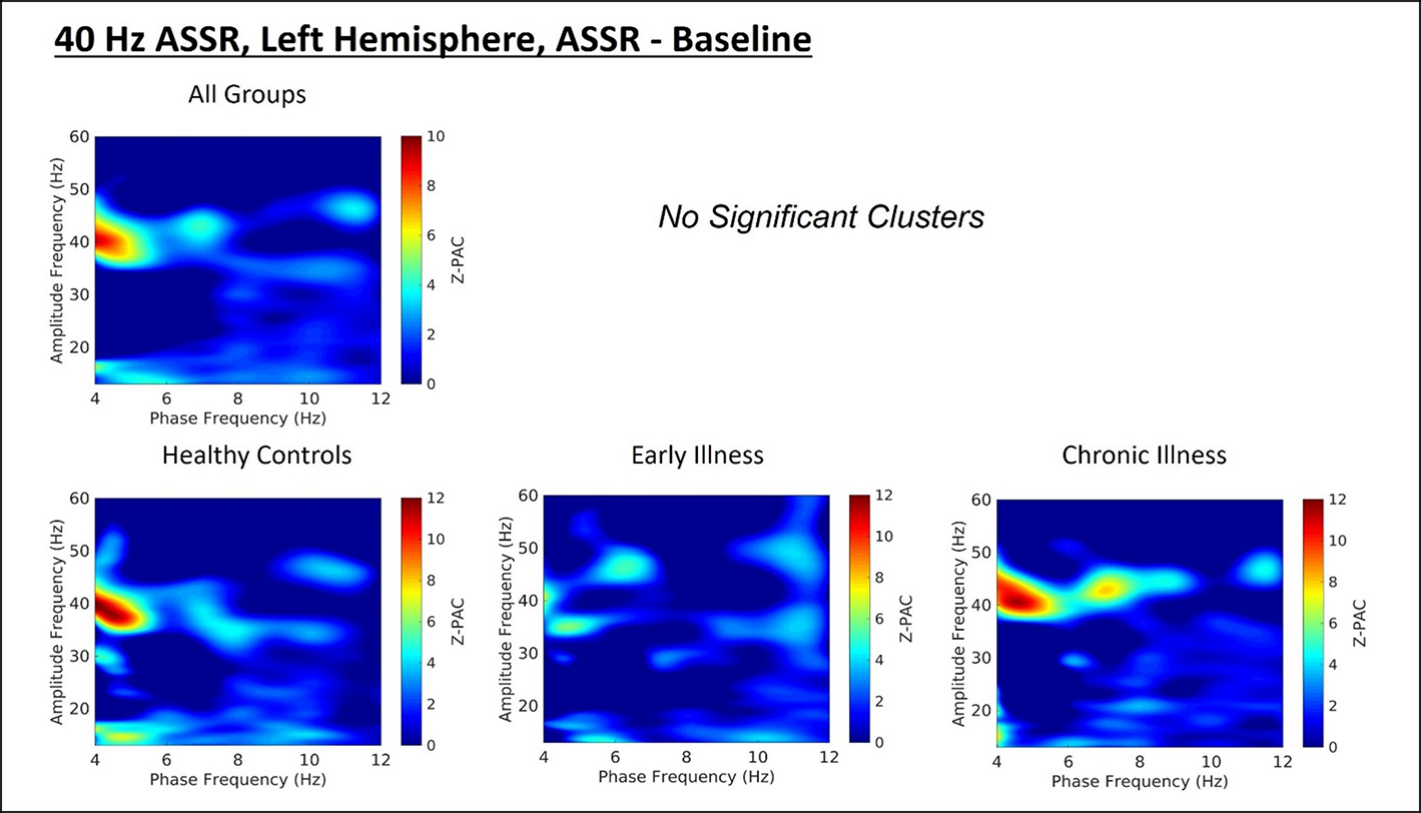
(Top) Left hemisphere Phase-amplitude coupling T-maps demonstrating differences between stimulation and baseline periods of the trial, with subjects pooled across groups. (Bottom) Left hemisphere PAC compared between groups.

**Figure 4:**
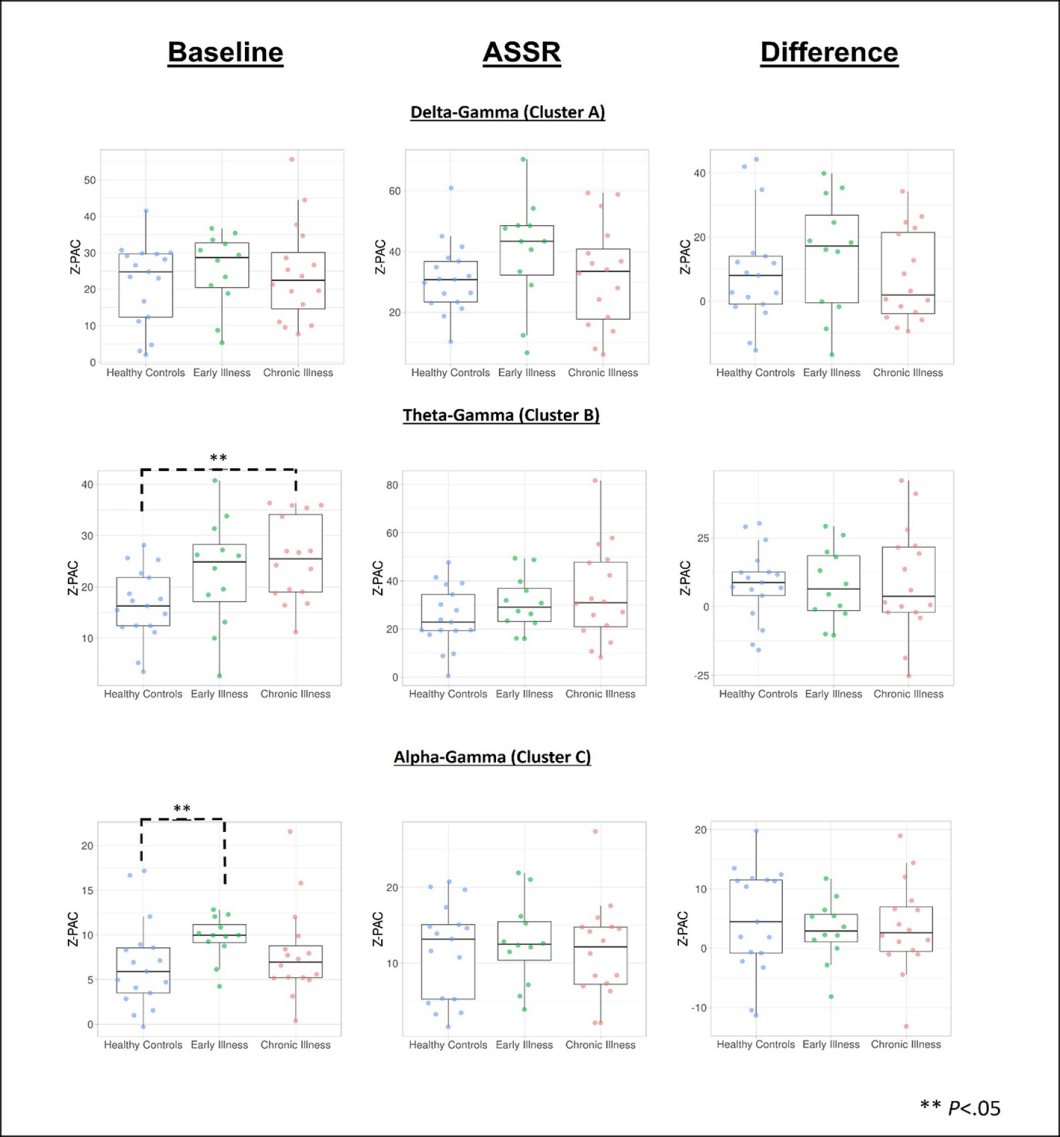
Data visualization of cluster PAC information created using the PlotsOfData web app for data visualization (67). **Denotes significance at *p < .05*.

Across all subjects there was a significant positive correlation between baseline PAC and high-frequency oscillation amplitude for the alpha-gamma coupling (r = 0.38, p = 0.01), as well as for low-frequency oscillation amplitude (r = 0.42, p = 0.004). Stimulus period PAC was not correlated with high- or low-frequency oscillation amplitudes. When this was tested at the individual group level we found that there was a significant positive correlation between alpha PAC and high-frequency oscillation amplitude in both the baseline (r = 0.64, p = 0.006) and stimulation periods (r = 0.72, p = 0.001) for healthy controls. There was no relationship between PAC and high-frequency oscillation amplitude for either patient group. There was no relationship between PAC and low-frequency oscillation amplitude for any of the groups. Our analysis of the rise-decay ratios for each low-frequency oscillation demonstrated that there was no significant differences in the presence of sinusoidal waveforms between the baseline and stimulus conditions (**Figures 2**–**4**).

### Wavelet Analysis

**Figure 5:**
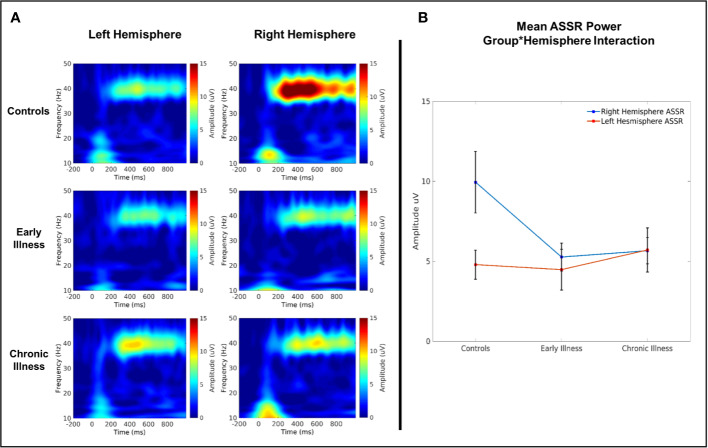
**(A)** Time–frequency plots of left and right auditory cortical 40 Hz ASSR amplitude. **(B)** 40 Hz evoked amplitude across groups.

In the 40-Hz condition, there was a significant interaction between group and hemisphere for 40 Hz amplitude (F(2, 40) = 4.08, p = 0.02, η^2^ = 0.17) (see **Figure 5**). Post hoc comparisons between left and right hemispheres on a per-group basis highlighted that there was a significantly greater right hemispheric power relative to left in HC (difference: 5.41 Uv ± 1.45 Uv; *p* = 0.001) but that this lateralization did not extend to EA and CR groups (*EA* difference: 1.01 Uv ± 1.7 Uv, *p* = 0.56; *CR* difference: −0.32 Uv ± 1.51 Uv, *p* = 0.83). Post-hoc comparisons between groups within each hemisphere highlighted a trend towards significantly elevated gamma power in the right hemisphere for the HC group relative to both the EA (difference: 4.97 Uv ± 2.1 Uv, *p* = 0.07) and CR groups (difference: 4.91 Uv ± 2.05 Uv, *p* = 0.06), but with no significant differences between the EA and CR groups. Analogous comparisons within the left hemisphere failed to find any significant differences between groups. A similar qualitative pattern occurred in the 40-Hz phase locking data; however, there were no significant effects or interactions. Amplitude and ITC data for the 30- and 20-Hz conditions also showed no main effects or interactions (see **Supplements 2** and **3**).

### Clinical Correlations

Based on the direction of our group findings we performed *post hoc* Spearman’s correlations to understand how variability in the severity of the illness, using the BACS and GAF scores, respectively, affected the distribution of our amplitude and PAC data. We corrected for multiple comparisons using an adjusted alpha value of 0.025. There were no significant correlations between PAC values or amplitude values with BACS or GAF scores.

## Discussion

Disturbances in gamma oscillations are considered to be a core feature of the pathophysiology that affects sensory and cognitive processing in schizophrenia. Investigations of gamma oscillations and PAC describe an interaction between multiple networks with different spatial profiles (68–71) where one network acts to rhythmically shift the membrane potential in the neurons that regulate local gamma oscillations (69, 72). The functional purpose of this interaction is believed to be the enhancement of neurocomputational efficiency *via* dynamic modulation of interneuron activity in a context dependent fashion. In the primate auditory cortex, there is evidence of theta/gamma PAC serving to optimize the processing of rhythmic inputs (73). In the context of schizophrenia therapeutics, it is pertinent to consider that focusing narrowly on the restoration of gamma oscillations without regard to their interactions with lower frequency activity may be insufficient to improve cognitive function. Previous studies of PAC in schizophrenia have reported a relative sparing of PAC relative to healthy controls (17, 27). However these studies are low in number and due to the methodological differences, it is difficult to conclude on findings.

Our aim was to study source localized PAC in the auditory cortex in schizophrenia patients at early and chronic stages of the disease to investigate the influences of disease progression and medication on PAC. Our findings were consistent with previous reports demonstrating an apparent sparing of ASSR-related PAC in patients at both early and chronic stages of the illness, in contrast with impaired cortical gamma oscillation amplitudes and phase coherence. The distinction between the main effects of pathology on neurophysiological processes supports the notion that the generation and modulation of gamma oscillations are related to distinct circuitry, which appears to be differentially affected by schizophrenia neuropathology.

Although it is possible to relate the contrast between ASSR power and PAC findings in the auditory cortex to potential nuances of pathophysiology, it is necessary to consider the component processes. In particular, it will be useful to establish generators for the low- and high-frequency oscillations that are functionally relevant to the auditory cortex. The generators of low-frequency oscillations, especially theta rhythms are associated with somatostatin (SST)-expressing (74) and multipolar bursting-type GABA interneurons (75). The interaction between SST and pyramidal neurons may be responsible for impaired theta frequency activity found in schizophrenia patients (10) and could be implicated in impaired auditory N100 and MMN generation (10, 76). More recently, a MEG study found some evidence for theta oscillations to be involved in the generation of early signatures of auditory prediction error (77). For gamma oscillations, the evidence is plenty and points to impairments in synchrony within auditory pyramidal–parvalbumin (PV) neuron circuits (10, 78). Despite evidence of the individual components involved in PAC being dysfunctional in schizophrenia, it is possible that the mechanisms governing the interaction between these neural generators could be intact in the disease. Further study will be necessary to elucidate the cellular mechanisms that may remain intact and support coupling, even in the face of disturbed mechanisms that lead to impairments in the respective coupled rhythms.

The gap in our understanding of specific low-level aspects of PAC mechanisms means that conclusions regarding intact PAC in schizophrenia will need to be qualified by further considerations, such as regional specificity and task demands. PAC impairments may be altered in other cortical regions or in the context of active demands (79–81). Future studies could systematically vary cortical regions and task demands to elucidate this issue.

Our analysis of the evoked ASSR properties support previous findings of a reduction in right hemisphere lateralization of gamma power (82, 83). The right auditory cortex may be more responsive to pitch processing and sound periodicity as found by several imaging and lesion studies (84, 85). Interestingly, it has been reported that schizophrenia patients show pitch-change detection deficits (83), which extends to findings of poorer auditory perception in schizophrenia (10, 83, 86), and imply an absence of appropriate task-related neural activation (82). As described above, schizophrenia pathophysiology may involve diminished or reversed brain asymmetry neuroanatomically and neurophysiologically (87).

In theoretical models of PAC, the strength of PAC reflects the network activity regulating modulation at the point of measurement (in this case the auditory cortex), which is scaled relative to the information flow necessary for an operation to function (88). In a recent comprehensive investigation of whole brain connectivity patterns in schizophrenia, Vergara and colleagues (89) noted a reduction in global connectivity strength, as well as increased randomness (and lower connectivity) between cognitive and sensory domains in patients relative to controls. The mixture of findings from structural (10), functional (89, 90), and neurophysiological (15, 91) studies in schizophrenia suggests that findings of intact PAC with the ASSR may be due to the paradigm being insufficiently sensitive to actual functional impairments under more ecological conditions. While the ASSR can be modulated by attention (92), it is largely a passive response to an external periodically driven stimulus, whereas working memory tasks require more active engagement and engage endogenously generated oscillatory dynamics. In our experiment, we couched our stimulus presentation in the context of an oddball-counting task in attempts to control for attentional effects across conditions and groups. Thus, while attention was engaged, it was not the focus of investigation and the oscillatory dynamics were primarily driven exogenously (as evidenced by the entrained response being closely aligned with the driving frequency). Accordingly, the differences in endogenous vs. exogenous origins of oscillations, task demands and associated cortical regions, may all contribute to whether cross-frequency coupling is impaired or intact. To elucidate the functional and spatial profile of cross-frequency coupling in schizophrenia, future studies could systematically manipulate task demands across sensory and cognitive domains and include both passive and active elicitation of oscillatory dynamics.

### Strengths and Limitations

Relative to previous studies our experimental approach provides an improved spatial and spectral resolution with which to study sensory gamma PAC in schizophrenia. The reduction of spatial smearing compared to EEG (36) improves the accuracy with which local estimations of time frequency resolved power and phase can be estimated. In particular, the ability to accurately derive the phasic properties of oscillations is important for avoiding spurious coupling estimates, where the extent of the coupling is inflated by irregular patterns in the signal that can distort the derivation of the phase or amplitude time series.

In this study we chose to apply the mean vector length (MVL) method (56) to estimate PAC as it would produce findings that were directly comparable with the study by Kirihara and colleagues (27). The estimation of PAC using the MVL approach measures the uniformity of vector points around zero, where deviation (long vector length) equates to phasic modulation of the amplitude (93). While this makes for a sensitive measure of detecting coupling that is robust to random noise; it is also sensitive to the absolute amplitude of high-frequency signal (35, 93, 94). To combat this we followed guidelines for appropriate estimation of PAC described in Seymour et al. (35), which emphasizes a need for both surrogate and baseline correction when possible. In the baseline period, we observed a trend for greater PAC strength in patients relative to controls that was also positively correlated with the amplitude of the low- and high-frequency time series signals. Both patient groups demonstrated potential signal to noise ratio declines relative to the control group; however, due to their strict occurrence within the baseline period they are unlikely to represent a confounding factor in the outcome of our analysis.

The study has a few limitations, including the subjects recruited, that had modest sample sizes and some gender imbalance across the groups. Although schizophrenia disproportionately affects males there is evidence of gender differences in neural activity associated with gamma oscillations (95) that should be considered in a larger, more representative study sample. In a similar vein, the effects of medication over time might contribute to a greater proportion of the variance than the current design is powered to systematically investigate.

### Conclusion

In this study, we investigated the hierarchical organization of gamma oscillations within the auditory cortex of patients with schizophrenia. Our findings are consistent with previous reports of intact cross-frequency coupling and further demonstrated that this pattern is present in both early and chronic stages of the illness. We believe that PAC should continue to be explored as a biomarker for schizophrenia due to its ability to index a component of oscillatory dynamics distinct from the basic mechanisms of those enabling high-frequency cortical oscillations and potential for tracking and predicting functional outcome. Future studies will more systematically investigate the differential impacts and interactions of task, cortical region, and illness phase (spanning prodromal to chronic and late phase illness).

## Data Availability Statement

Datasets are available upon request. The raw data supporting the conclusions of this article will be made available by the authors, without undue reservation, to any qualified researcher.

## Ethics Statement

The studies involving human participants were reviewed and approved by University of Texas Health Science Center at Houston Institutional Review Board. The patients/participants provided their written informed consent to participate in this study.

## Author Contributions

RC, NP, and CW designed the study. NM and NR conducted data collection. NM and NR designed and conducted the data pre-processing, NM conducted the feature extraction and statistical analysis. NM and NR wrote the first draft of the manuscript. All authors contributed to manuscript revision, read, and approved the submitted version.

## Funding

This work was supported by the R21 Grant No. MH103541-01 from the National Institute of Health.

## Conflict of Interest

The authors declare that the research was conducted in the absence of any commercial or financial relationships that could be construed as a potential conflict of interest.
