# Supplementary material for: Intact Auditory Cortical Cross-Frequency Coupling in Early and Chronic Schizophrenia
Source: Front Psychiatry. 2020 Jun 4;11:507. doi: 10.3389/fpsyt.2020.00507 (PMC7287164; doi:10.3389/fpsyt.2020.00507)
Supplement: Supplementary file 1 [file DataSheet_1.docx]

**Supplemental Information**


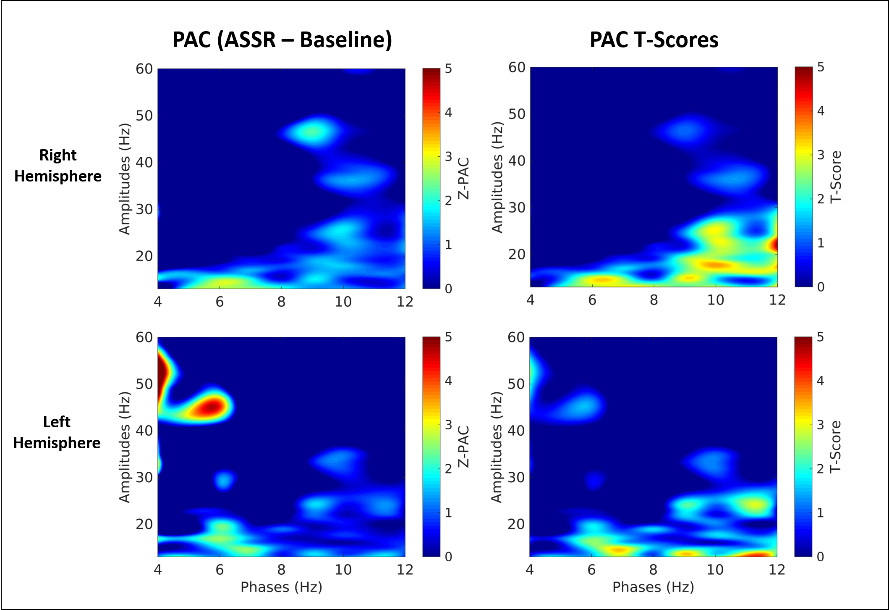
**Supplement 1 – Phase-Amplitude Coupling**

**Figure S1,** Left and right hemisphere PAC during the evoked 30 Hz ASSR condition**.**


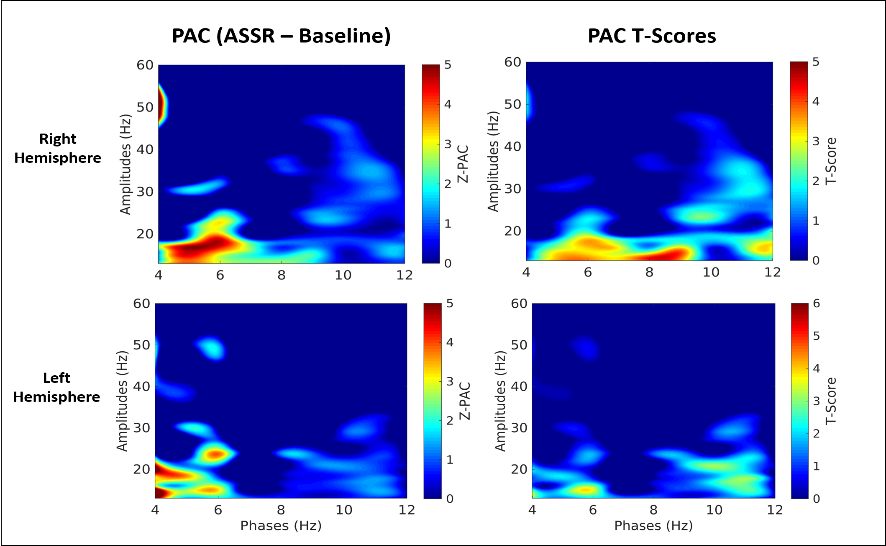


**Figure S2,** Left and right hemisphere PAC during the evoked 20 Hz ASSR condition**.**


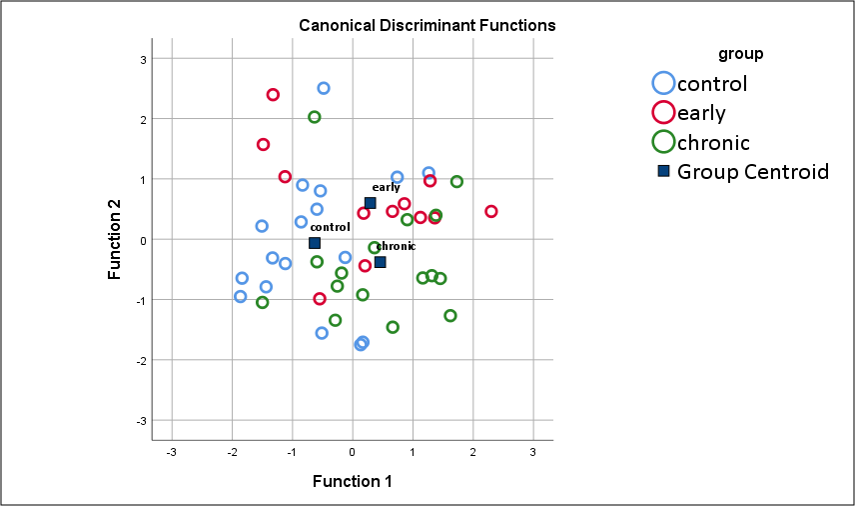

**Figure S3,** Distribution of the canonical discriminant functions described in the posthoc follow-up to the Multivariate Analysis of Covariance conducted on the baseline time-period PAC data.

**Supplement 2 – ASSR Wavelet Power**

**
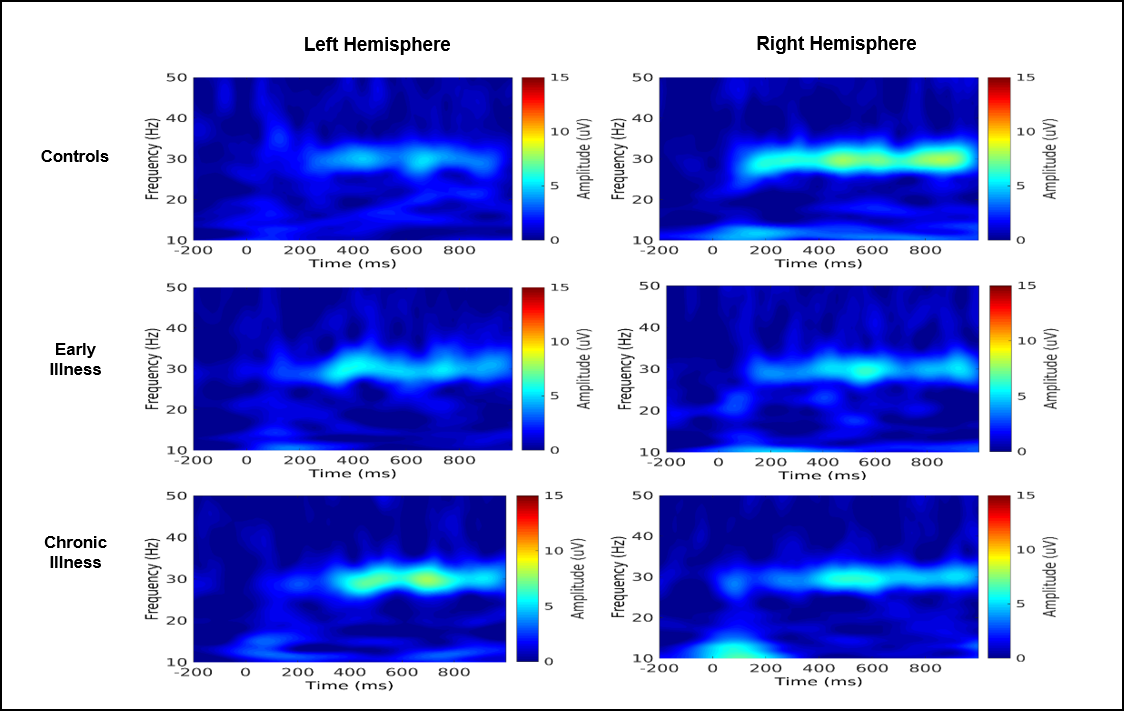
Figure S4,** Left and right hemisphere time-frequency representations of the evoked wavelet data during the 30 Hz ASSR condition.

**
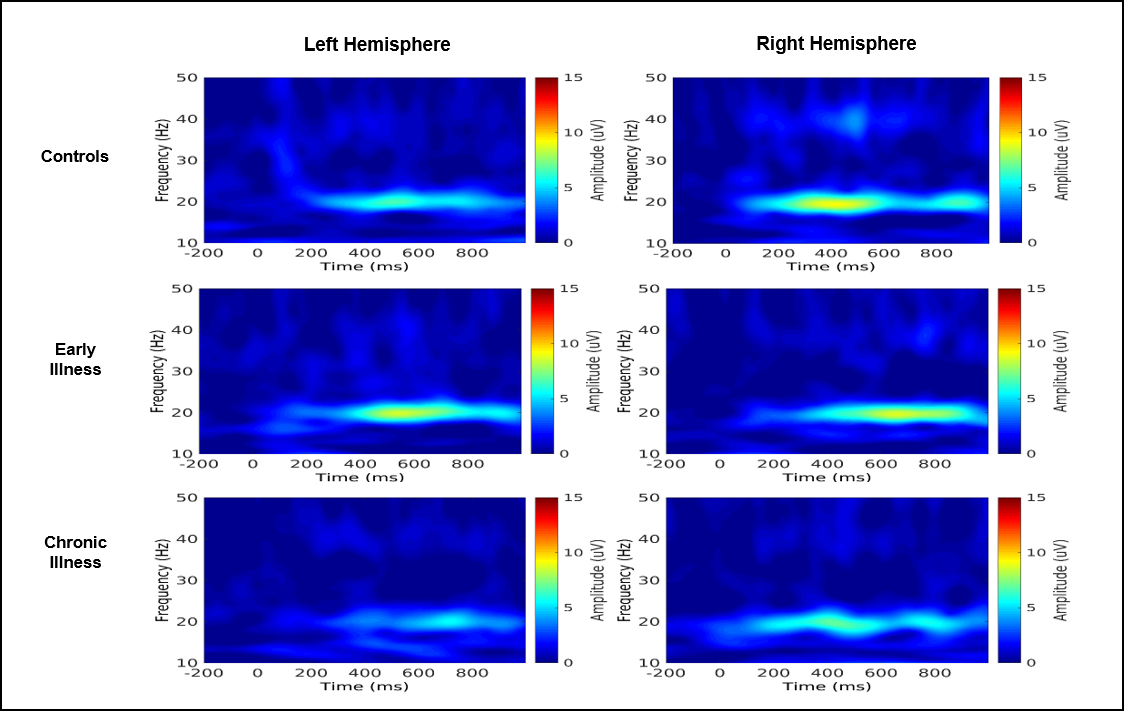
Figure S5,** Left and right hemisphere time-frequency representations of the evoked wavelet data during the 20 Hz ASSR condition.

**Supplement 3 – ASSR Wavelet Inter-Trial Phase Coherence**

**
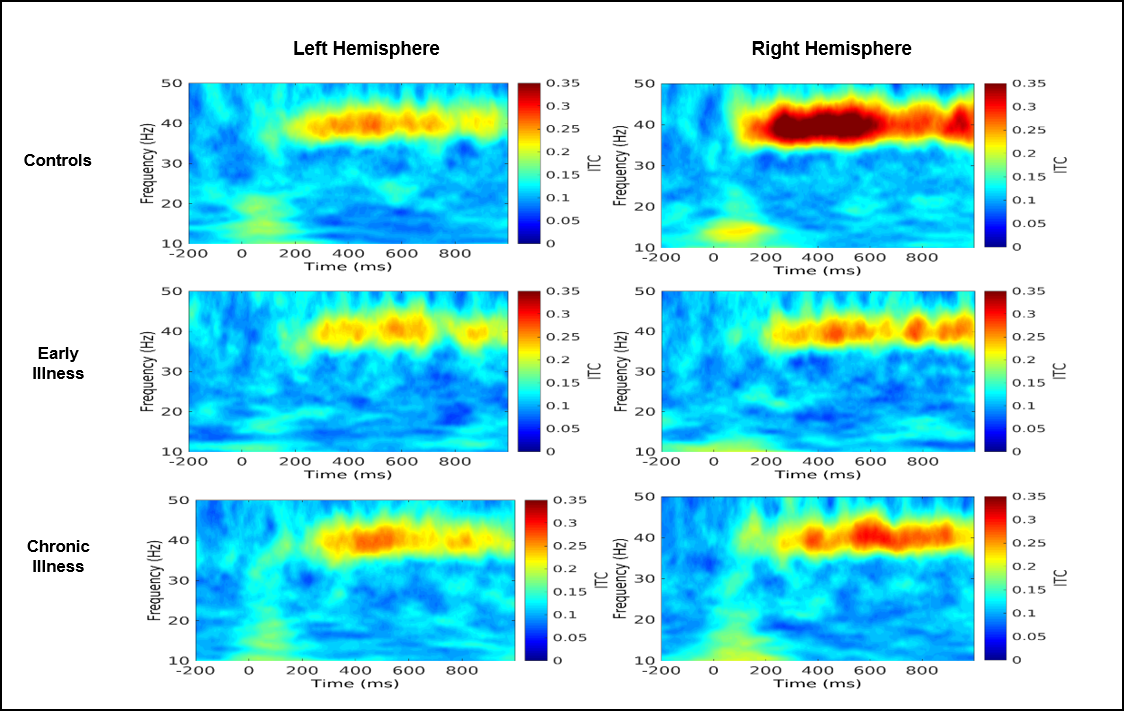
Figure S6,** Left and right hemisphere time-frequency inter-trial phase coherence during the evoked 40 Hz ASSR condition.

**
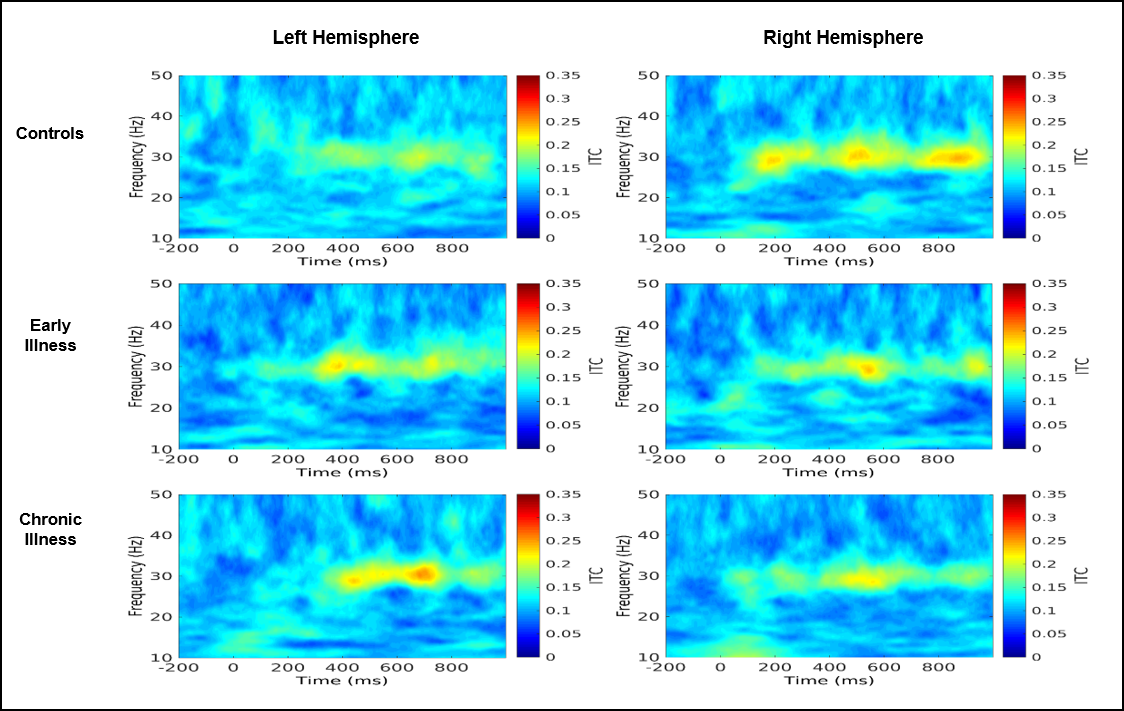
Figure S7,** Left and right hemisphere time-frequency inter-trial phase coherence during the evoked 30 Hz ASSR condition.

**
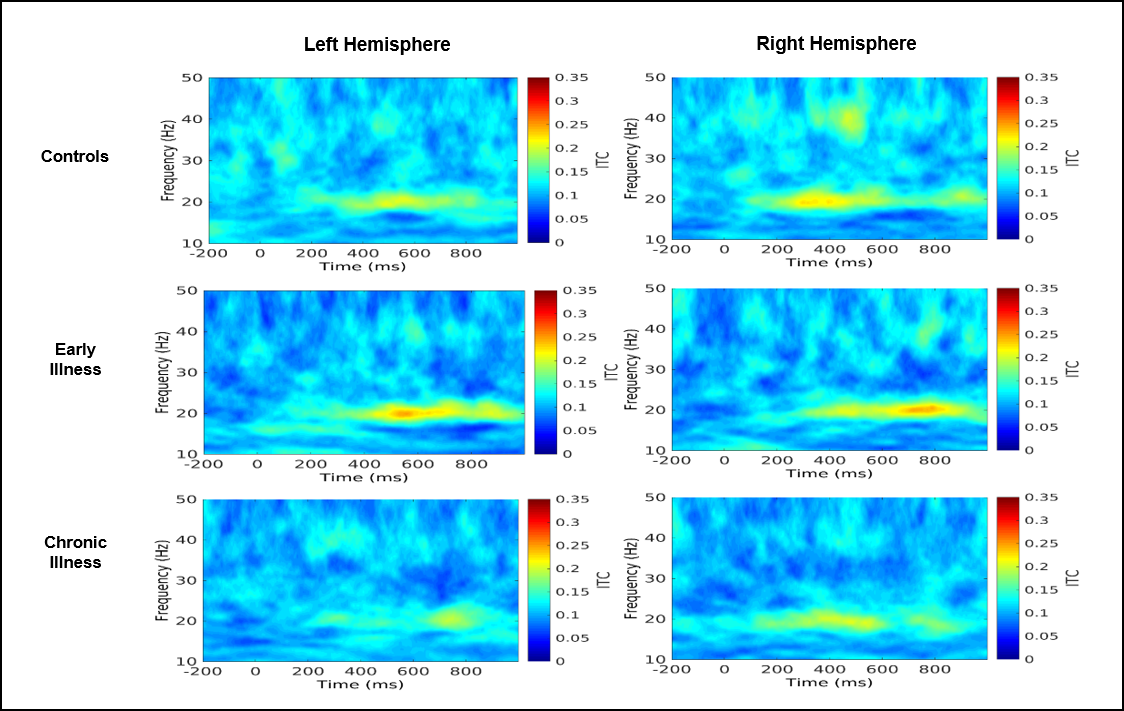
Figure S8,** Left and right hemisphere time-frequency inter-trial phase coherence during the evoked 20 Hz ASSR condition**.**
